# Interpretable machine learning models based on CT radiomics for predicting chemoradiotherapy response in rectal cancer

**DOI:** 10.1186/s12880-026-02269-4

**Published:** 2026-03-20

**Authors:** Jianfeng Li, Shunping Huang, Yuan Peng, Haiyan Peng, Wenyou Hu, Meijuan Sun, Yuemei Dong, Nan Zhao, Zhaoxia Li, Fu Jin, Ning Wang

**Affiliations:** 1 Radiation Physics Center, Chongqing University Cancer Hospital, Chongqing, China; 2 Department of Cancer Center, The Second Affiliated Hospital of Chongqing Medical University, Chongqing, China; 3 Department of Oncology, Hebei Guan County People’s Hospital, Hebei, China; 4 Department of Oncology, PLA Rocket Force Characteristic Medical Center, Beijing, China

**Keywords:** Radiomics, Rectal cancer, Machine learning, SHAP, Neoadjuvant chemoradiotherapy

## Abstract

**Background:**

Accurate prediction of response to neoadjuvant chemoradiotherapy (nCRT) in patients with locally advanced rectal cancer (LARC) is essential for optimizing treatment decisions. This study aimed to develop interpretable machine learning models based on computed tomography (CT) radiomics and clinical biomarkers to predict nCRT efficacy.

**Methods:**

A total of 272 patients with pathologically confirmed LARC were retrospectively included and divided into training (*n* = 156), internal validation (*n* = 67), and external validation (*n* = 49) sets. Radiomics features were extracted from pretreatment contrast-enhanced CT images. A radiomics score (R-score) was constructed from 10 LASSO-selected features with high reproducibility (intraclass correlation coefficient > 0.75). Clinical variables including carcinoembryonic antigen (CEA) and carbohydrate antigen 19 − 9 (CA19-9) were incorporated. Logistic regression, support vector machine, random forest, decision tree, and XGBoost algorithms were used to develop predictive models. Model performance was assessed by area under the receiver operating characteristic curve (AUC), calibration curve, and decision curve analysis (DCA). SHapley Additive exPlanations (SHAP) were used to interpret model output.

**Results:**

In the test cohorts, the combined model using the XGBoost algorithm outperformed clinical-only and imaging-only models, achieving AUCs of 0.844 (internal validation) and 0.800 (external validation). The R-score was significantly higher in responders than in non-responders (*P* < 0.05 across all datasets). DCA demonstrated superior clinical net benefit of the combined model across threshold probabilities. SHAP analysis confirmed R-score as the most influential predictor of response.

**Conclusions:**

The XGBoost-based combined model integrating CT radiomics and clinical biomarkers demonstrated robust performance and good interpretability in predicting nCRT response in LARC patients. This approach may support individualized treatment planning and risk stratification in clinical practice.

**Supplementary Information:**

The online version contains supplementary material available at 10.1186/s12880-026-02269-4.

## Introduction

Colorectal cancer is one of the most common malignancies worldwide, ranking third in incidence and showing a particularly rapid increase in developing countries [1, 2]. For patients with locally advanced rectal cancer (LARC), neoadjuvant chemoradiotherapy (nCRT) has become the standard treatment, offering advantages such as tumor downstaging, improved resectability, and prolonged survival [3, 4]. However, treatment response to nCRT varies greatly among individuals. Only a minority of patients achieve pathologic complete response, while nonresponders not only fail to benefit but also suffer from unnecessary drug-related toxicities [5, 6]. Thus, accurately predicting response to nCRT before treatment remains a critical clinical need for achieving individualized treatment strategies.

At present, therapeutic response is typically assessed after nCRT using imaging examinations such as MRI or CT and postoperative tumor regression grading. Traditional imaging assessments largely rely on tumor size changes (e.g., RECIST criteria), which are inherently limited by delayed evaluation and difficulties in distinguishing between residual tumor and treatment-induced fibrosis [7]. Moreover, imaging interpretation is susceptible to radiologist expertise, resulting in subjective variability [8]. Consequently, there is an urgent need for reliable methods capable of predicting nCRT response in LARC patients at the pretreatment or early treatment stage to support optimized clinical decision-making.

Radiomics, a high-throughput quantitative image analysis technique, enables extraction of a large number of features from conventional medical images, thereby reflecting underlying tumor heterogeneity [9]. Combined with machine learning and deep learning approaches, radiomics has demonstrated significant potential in predicting treatment response and prognosis across multiple cancers [10, 11]. However, the “black-box” nature of many complex models often limits interpretability and hinders clinical adoption [12]. To address this limitation, SHapley Additive exPlanations (SHAP) has been introduced as a powerful interpretability tool. SHAP provides both local and global explanations for machine learning predictions, intuitively visualizing feature contributions and enhancing transparency, which may improve acceptance in clinical practice [13].

Therefore, the aim of this study was to develop machine learning models based on pretreatment contrast-enhanced CT radiomics features to predict nCRT response in patients with LARC. Furthermore, SHAP was incorporated to provide model interpretability and visualize the impact of key features, with the goal of delivering a clinically reliable and explainable prediction tool.

## Materials and methods

### Study sample

This retrospective study was conducted in accordance with the principles of the Declaration of Helsinki and was approved by the institutional review boards of all participating centers (ethics approval numbers: CZLS2025145-A at Chongqing University Cancer Hospital and [2022] 651 at the Second Affiliated Hospital of Chongqing Medical University). Due to the retrospective nature of the study, the requirement for written informed consent was waived by the ethics committees.

A total of 290 patients with LARC who received nCRT between February 2021 and August 2023 at Chongqing University Cancer Hospital and 68 patients treated between January 2022 and June 2023 at the Second Affiliated Hospital of Chongqing Medical University were retrospectively included.

The inclusion criteria were as follows: *(a)* pathologically and radiologically confirmed diagnosis of LARC; *(b)* total mesorectal excision (TME) performed after completion of nCRT; and *(c)* availability of high-quality pretreatment CT scans with clear tumor visualization. The exclusion criteria were as follows: *(a)* inadequate CT image quality or unidentifiable tumor regions (e.g., severe artifacts); *(b)* failure to complete the planned nCRT regimen; and *(c)* prior treatment for rectal cancer (radiotherapy, surgery, or systemic therapy) that could confound study results.

Patients from Chongqing University Cancer Hospital were randomly stratified into training and internal validation sets at a 7:3 ratio, while patients from the Second Affiliated Hospital of Chongqing Medical University were used as an external validation set.

### Clinical and imaging data collection

Clinical data collected included patient sex, age, body mass index (BMI), and tumor regression grade (TRG). TRG was assessed based on the American Joint Committee on Cancer (AJCC) criteria and used to classify patients as responders (TRG 0–1) or non-responders (TRG 2–3). Additional clinical parameters included serum carcinoembryonic antigen (CEA) and carbohydrate antigen 19 − 9 (CA19-9) levels, clinical tumor/node staging, and pathological differentiation (well, moderate, or poor).

All patients underwent contrast-enhanced CT targeting the radiotherapy planning region. CT images were interpreted by two board-certified radiologists with more than 5 years of experience in abdominal imaging and further reviewed by a senior radiologist with over 10 years of experience to ensure consistency.

### CT imaging protocol

All contrast-enhanced CT scans were performed using a Philips Brilliance Big Bore CT scanner (Philips Healthcare, Eindhoven, the Netherlands). Patients were scanned in the supine position with head-first orientation. The scanning range extended from the dome of the diaphragm to the inferior margin of the pubic symphysis and included axial, sagittal, and coronal planes. Scanning parameters were as follows: tube voltage, 120 kV; tube current, 260–300 mA; field of view, 600 × 600 mm; matrix size, 1024 × 1024; reconstruction kernel, standard; and slice thickness, 1.5 mm. Iopromide was administered as the contrast agent at an injection rate of 3–5 mL/s.

### nCRT protocol

All patients received a standardized nCRT regimen. Radiotherapy consisted of long-course pelvic radiation covering the primary rectal tumor and regional lymphatic drainage areas, including the mesorectal, presacral, internal iliac, and obturator nodal regions. A total dose of 50.4 Gy was delivered in 28 fractions, with five sessions per week at 1.8 Gy per fraction. Intensity-modulated radiation therapy (IMRT) was employed to ensure precise dose delivery to the target volume while minimizing radiation exposure to surrounding normal tissues.

Concurrent chemotherapy was administered with oral capecitabine (825 mg/m², twice daily), used as a radiosensitizer throughout the entire radiotherapy course without interruption. The dosing schedule was adjusted as needed based on individual patient tolerance, including bone marrow suppression and gastrointestinal side effects.

### Image preprocessing and feature extraction

Pretreatment CT images were manually segmented by two radiation oncologists with 10 and 12 years of experience in oncologic imaging, respectively. The region of interest (ROI) was defined on the axial slice containing the largest tumor cross-section and adjacent slices, excluding intraluminal contents, gas, and surrounding soft tissues. All segmentations were reviewed and confirmed by a senior radiologist. In cases of disagreement, consensus was reached through discussion.

Radiomic feature extraction was performed using the PyRadiomics package in Python (version 3.1; https://pyradiomics.readthedocs.io). A total of 107 features were extracted, including: (1) 14 shape features; (2) 18 first-order statistical features; and (3) 75 texture features, consisting of 24 gray-level co-occurrence matrix (GLCM) features, 16 gray-level run length matrix (GLRLM) features, 16 gray-level size zone matrix (GLSZM) features, 14 gray-level dependence matrix (GLDM) features, and 5 neighborhood gray-tone difference matrix (NGTDM) features.

All features were Z-score normalized before model development. PyRadiomics was used with default settings to ensure consistency and reproducibility across patients.

### Feature selection and model construction

Radiomic feature selection was conducted in three steps: *(a)* reproducibility was evaluated using the intraclass correlation coefficient (ICC), and only features with ICC > 0.75 were retained; *(b)* least absolute shrinkage and selection operator (LASSO) regression was applied to remove redundant and collinear features and select independent predictors; and *(c)* a multivariate logistic regression model was constructed using the selected features. Prediction probabilities were normalized to generate a radiomics score (R-score) ranging from 0 to 1.

In the training set, clinical variables were first screened using univariate logistic regression to identify factors significantly associated with nCRT response (*P* < 0.05). Significant variables were then incorporated into a multivariate logistic regression model to determine independent predictors and construct a clinical model. To enhance predictive performance, the R-score was combined with significant clinical variables to develop multiple machine learning models, including logistic regression (LR), random forest (RF), support vector machine (SVM), decision tree (DT), and extreme gradient boosting (XGBoost). The model with the highest area under the curve (AUC) was selected for further evaluation in the internal and external validation sets.

Additionally, the best-performing model constructed in the training set was interpreted using SHAP to assess the contribution of each feature to the model’s predictions.

### Statistical analysis

The normality of continuous variables was assessed using the Shapiro–Wilk test, and homogeneity of variance was evaluated using Levene’s test. All variables satisfied the assumptions of normal distribution and equal variance. Between-group comparisons for continuous variables were performed using independent-samples t tests and reported as mean ± standard deviation. Categorical variables were expressed as counts and percentages, and compared using the chi-square test or Fisher’s exact test, as appropriate.

Univariate and multivariate logistic regression analyses were used to identify independent predictors of nCRT response. A nomogram was constructed based on these predictors. Model discrimination was evaluated using the area under the receiver operating characteristic (ROC) curve (AUC), calibration was assessed via calibration curves, and clinical utility was evaluated using decision curve analysis (DCA). All statistical tests were two-sided, and *P* < 0.05 was considered statistically significant. Analyses were performed using R software (version 4.3.1) and Python (version 3.8.2).

## Results

### Baseline characteristics of patients

A total of 358 patients were initially screened from two participating centers, among whom 86 were excluded based on the eligibility criteria. The final study cohort consisted of 223 patients from Chongqing University Cancer Hospital, including 156 patients assigned to the training set (mean age, 57.21 ± 10.27 years) and 67 patients to the internal validation set (mean age, 56.67 ± 11.34 years). Additionally, 49 patients from the Second Affiliated Hospital of Chongqing Medical University were included in the external validation set (mean age, 55.22 ± 9.27 years).

The proportion of patients achieving pCR was 21.8% (34/156) in the training set, 20.9% (14/67) in the internal validation set, and 40.8% (20/49) in the external validation set. Significant differences were observed across the three cohorts in CEA, N stage, and treatment response (all *P* < 0.05) (Table 1).

**Table 1:** General characteristics and intergroup differences across cohorts

Characteristics	Training Set (*n* = 156)	Internal Validation Set (*n* = 67)	External Validation Set (*n* = 49)	*P* Value
**Age (years)**	57.21 ± 10.27	56.67 ± 11.34	55.22 ± 9.27	0.478
**BMI**	23.06 ± 3.02	23.58 ± 2.88	23.68 ± 2.66	0.114
**Sex**				
Male	126 (80.8)	51 (76.1)	35 (71.4)	
Female	30 (19.2)	16 (23.9)	14 (28.6)	
**CA19-9 (kU/L)**	25.63 ± 62.43	17.72 ± 15.84	39.19 ± 95.20	0.106
**CEA (ng/mL)**	6.55 ± 8.37	6.33 ± 6.49	20.17 ± 51.26	0.005
**T stage**				0.912
T2	4 (2.6)	1 (1.5)	2 (4.1)	
T3	96 (61.5)	42 (62.7)	28 (57.1)	
T4	56 (35.9)	24 (35.8)	19 (38.8)	
**N stage**				0.040
N0	37 (23.7)	10 (14.9)	6 (12.2)	
N1	60 (38.5)	24 (35.8)	17 (34.7)	
N2	48 (30.8)	30 (44.8)	26 (53.1)	
N3	11 (7.0)	3 (4.5)	0 (0)	
**Tumor location**				0.068
Low	97 (62.2)	40 (59.7)	22 (44.9)	
Mid	55 (35.3)	21 (31.3)	23 (46.9)	
High	4 (2.6)	6 (9.0)	4 (8.2)	
**Tumor-differentiation grade**				0.263
Poorly	39 (25.0)	19 (28.4)	7 (14.3)	
Moderately	101 (64.7)	42 (62.7)	33 (67.3)	
Well	16 (10.3)	6 (9.0)	9 (18.4)	
**Treatment response**				0.018
Responders	34 (21.8)	14 (20.9)	20 (40.8)	
Non-Responders	122 (78.2)	53 (79.1)	29 (59.2)	

Note.—Except where indicated, data are numbers of patients, with percentages in parentheses

### Comparison of characteristics between responders and non-responders

In the training set, significant differences were observed between Responders and Non-Responders in several clinical characteristics, including CA19-9 (*p* < 0.001), CEA (*p* = 0.019), and tumor differentiation grade (*p* < 0.001). No significant differences were found in other variables such as age, BMI, sex, T stage, N stage, and tumor location (Table S1).

In the internal validation set, CEA (*p* = 0.005) and tumor differentiation grade (*p* < 0.001) also showed significant differences between the two groups, while all other variables remained nonsignificant (Table S2).

In the external validation set, only CA19-9 (*p* = 0.021) and CEA (*p* = 0.013) demonstrated statistically significant differences, with no significant group differences observed in the remaining clinical variables (Table S3).

### R-score construction

**Fig. 1:**
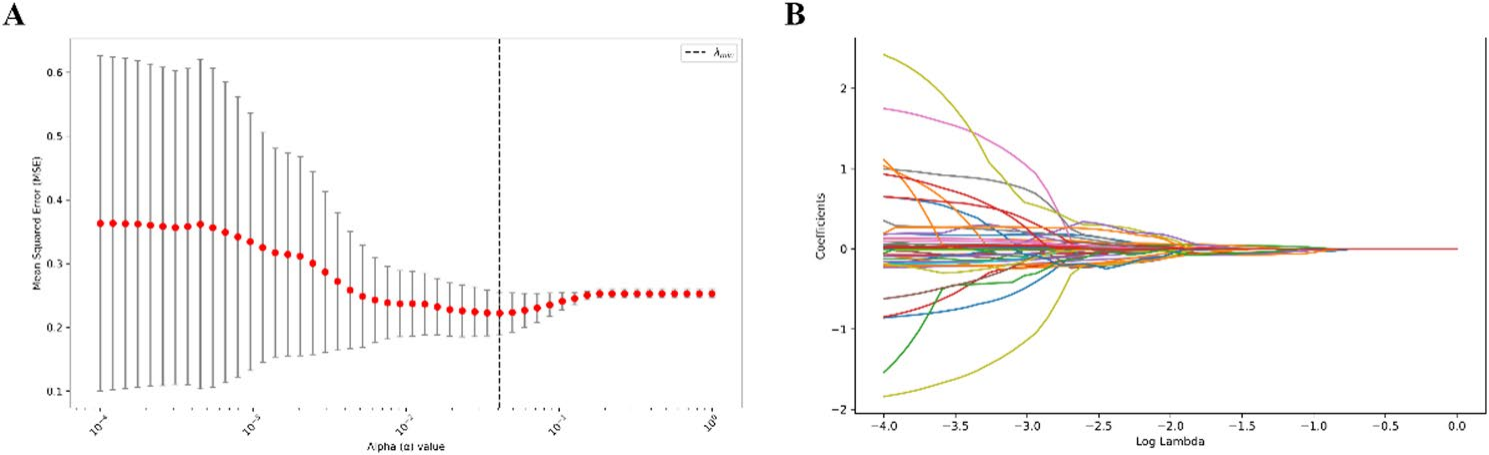
Feature Selection Using LASSO Regression in the Training Set. (**A**) Ten-fold cross-validation was performed to determine the optimal value of the regularization parameter (λ) in the LASSO regression model. The minimum mean squared error (MSE) was achieved at λ = 0.041. (**B**) Coefficient profiles of the 88 radiomic features as a function of the log(λ) value. A total of 10 non-zero features were selected and linearly combined with their respective LASSO coefficients to generate the radiomics score (R-score)

Among the 107 extracted radiomic features, 88 demonstrated good reproducibility with an ICC > 0.75. Based on these 88 features, LASSO regression was performed for feature selection, yielding an optimal regularization parameter of λ = 0.041 (Fig. 1A). A total of 10 features with non-zero coefficients were ultimately selected (Fig. 1B), and a weighted combination of these features was used to construct a composite radiomics score (R-score).

**Fig. 2:**
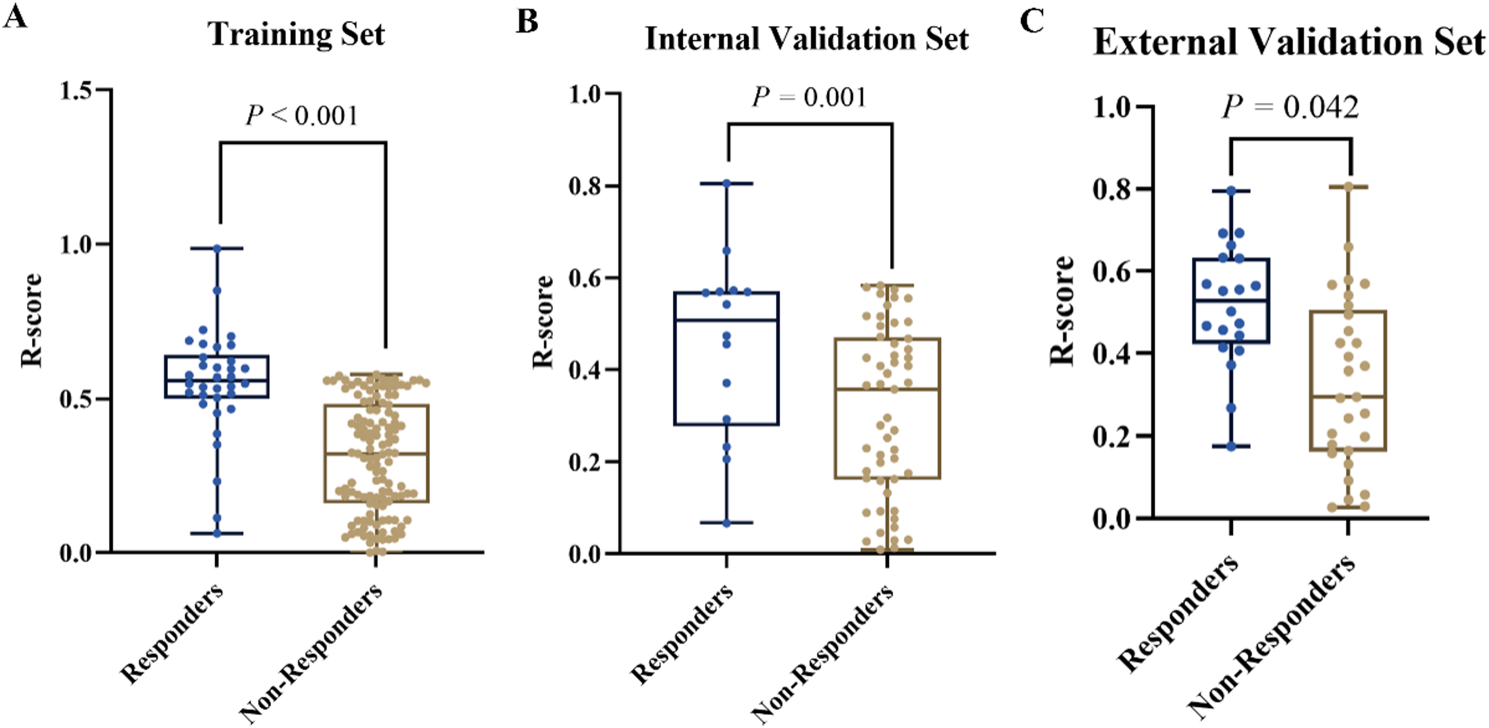
Comparison of R-scores Between Responders and Non-Responders in Three Sets. Box plots show the distribution of radiomics scores (R-scores) between Responders and Non-Responders in the (**A**) training set, (**B**) internal validation set, and (**C**) external validation set. R-scores were significantly higher in Responders across all cohorts (t-test: *P* < 0.001, *P* = 0.001, and *P* = 0.042, respectively)

The R-score was significantly higher in responders than in non-responders in the training set (*P* < 0.001), and this trend was consistently observed in both the internal validation set (*P* = 0.001) and the external validation set (*P* = 0.042) (Fig. 2).

### Variables associated with nCRT response

In the training set, univariate logistic regression analysis showed that CA19-9 (OR = 0.895, 95% CI: 0.840–0.954, *P* = 0.001), CEA (OR = 0.874, 95% CI: 0.782–0.977, *P* = 0.018), T4 stage (vs. T2, OR = 0.386, *P* = 0.040), moderate differentiation (vs. poor differentiation, OR = 0.817, *P* = 0.043), well differentiation (OR = 0.730, *P* = 0.039), and the R-score (OR = 5.983, 95% CI: 2.880–12.426, *P* < 0.001) were significantly associated with nCRT response.

Multivariate logistic regression further identified CA19-9 (OR = 0.912, 95% CI: 0.855–0.967, *P* = 0.026), CEA (OR = 0.931, 95% CI: 0.869–0.971, *P* = 0.031), and R-score (OR = 5.131, 95% CI: 2.261–10.186, *P* < 0.001) as independent predictors (Table 2).

**Table 2:** Univariable and multivariable logistic regression analysis of characteristics associated with nCRT response in the training set

Characteristic	Univariable		Multivariable	
	OR (95% CI)	*P* Value	OR (95% CI)	*P* Value
**Age (years)**	0.994 (0.958–1.031)	0.734		
**BMI**	1.068 (0.943–1.210)	0.303		
**Sex**				
Male	Reference			
Female	0.492 (0.159–1.524)	0.219		
**CA19-9 (kU/L)**	0.895 (0.840–0.954)	0.001	0.912 (0.855–0.967)	0.026
**CEA (ng/mL)**	0.874 (0.782–0.977)	0.018	0.931 (0.869–0.971)	0.031
**T stage**				
T2	Reference			
T3	1.403 (0.628–3.138)	0.409	1.211 (0.719–3.091)	0.434
T4	0.386 (0.156–0.956)	0.040	0.491 (0.264–1.134)	0.196
**N stage**				
N0	Reference			
N1	1.157 (0.533–2.510)	0.713		
N2	0.631 (0.262–1.518)	0.304		
N3	0.339 (0.042–2.749)	0.311		
**Tumor location**				
Low	Reference			
Mid	0.595 (0.255–1.385)	0.228		
High	1.202 (0.121–0.279)	0.875		
**Tumor-differentiation grade**				
Poorly	Reference			
Moderately	0.817 (0.649–0.981)	0.043	0.864 (0.781–1.216)	0.151
Well	0.730 (0.591–0.977)	0.039	0.793 (0.625–1.314)	0.133
**R-score**	5.983 (2.880-12.426)	< 0.001	5.131 (2.261–10.186)	< 0.001

Note.—Data in parentheses are 95% CI. CI = confidence interval, OR = odds ratio

Based on these results, a clinical model was developed using CA19-9 and CEA via logistic regression, and an imaging model was built using the R-score. To further enhance predictive performance, the three variables were combined and five machine learning algorithms were used to develop the combined model.

### Performance of different models

Among the five machine learning models constructed in the training set, the XGBoost model achieved the best performance, with an AUC of 0.858 (Figure S1). Therefore, XGBoost was selected as the combined model for predicting treatment response to nCRT in patients with LARC.

**Fig. 3:**
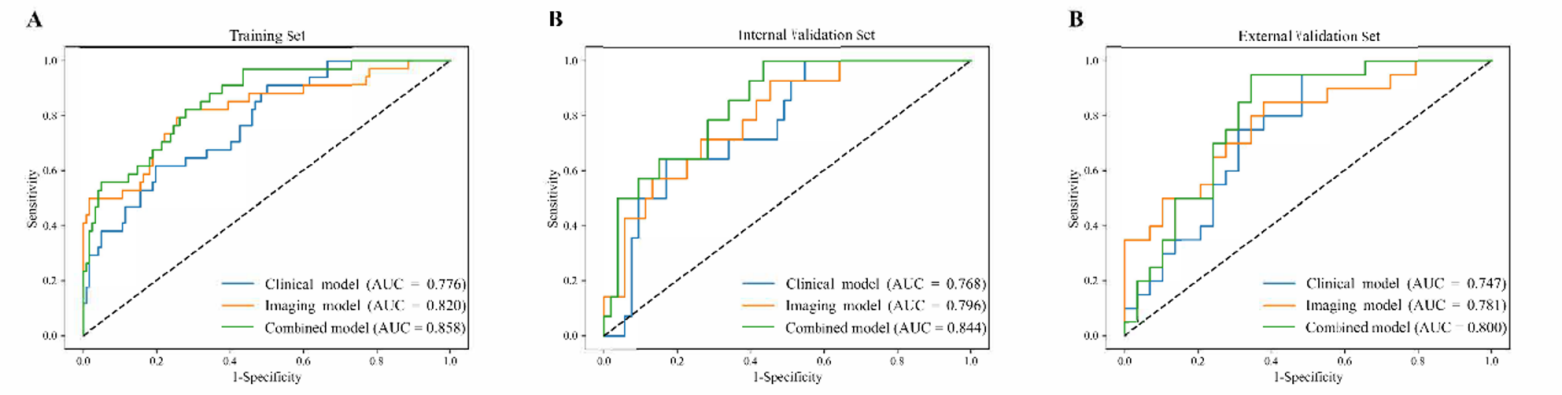
ROC Curves of Clinical, Imaging, and Combined Models Across All Sets. ROC curves of the clinical, imaging, and combined models in the training set (**A**), internal validation set (**B**), and external validation set (**C**). In all cohorts, the combined model consistently achieved the highest AUC, demonstrating superior predictive performance for nCRT response compared with the individual clinical or imaging models

In both the internal and external validation sets, the combined model demonstrated superior predictive performance compared to the clinical and imaging models (Delong test, all *P* < 0.05). The AUCs of the clinical model ranged from 0.747 to 0.768, while the imaging model achieved AUCs between 0.781 and 0.796. In contrast, the combined model yielded the highest AUCs in all datasets, with values 0.844 in the internal validation set, and 0.800 in the external validation set (Table 3; Fig. 3).

**Table 3:** Performances of models in predicting nCRT response

Model	Sets	AUC (95% CI)	Sensitivity (%)	Specificity (%)	*P* Value^†^
Clinical model	Training Set	0.776 (0.695–0.856)	61.8	80.3	/
	Internal Validation Set	0.768 (0.620–0.883)	64.3	83.0	/
	External Validation Set	0.747 (0.604–0.865)	95.0	51.7	/
Image model	Training Set	0.820 (0.727–0.904)	82.4	72.1	**0.026**
	Internal Validation Set	0.796 (0.677–0.907)	92.9	54.7	**0.037**
	External Validation Set	0.781 (0.635–0.898)	85.0	62.1	**0.031**
Combined model	Training Set	0.858 (0.791–0.924)	82.4	72.1	**< 0.001**
	Internal Validation Set	0.844 (0.729–0.934)	100.0	56.6	**< 0.001**
	External Validation Set	0.800 (0.665–0.918)	95.0	65.5	**< 0.001**

Note.—Except where indicated, data in parentheses are numbers of patients. AUC = area under the receiver operating characteristic curve, CI = confidence interval

^*^Data in parentheses are 95% CIs

^**†**^*P* value for comparison of the AUC with that of the clinical model (reference) in the corresponding cohort

**Fig. 4:**
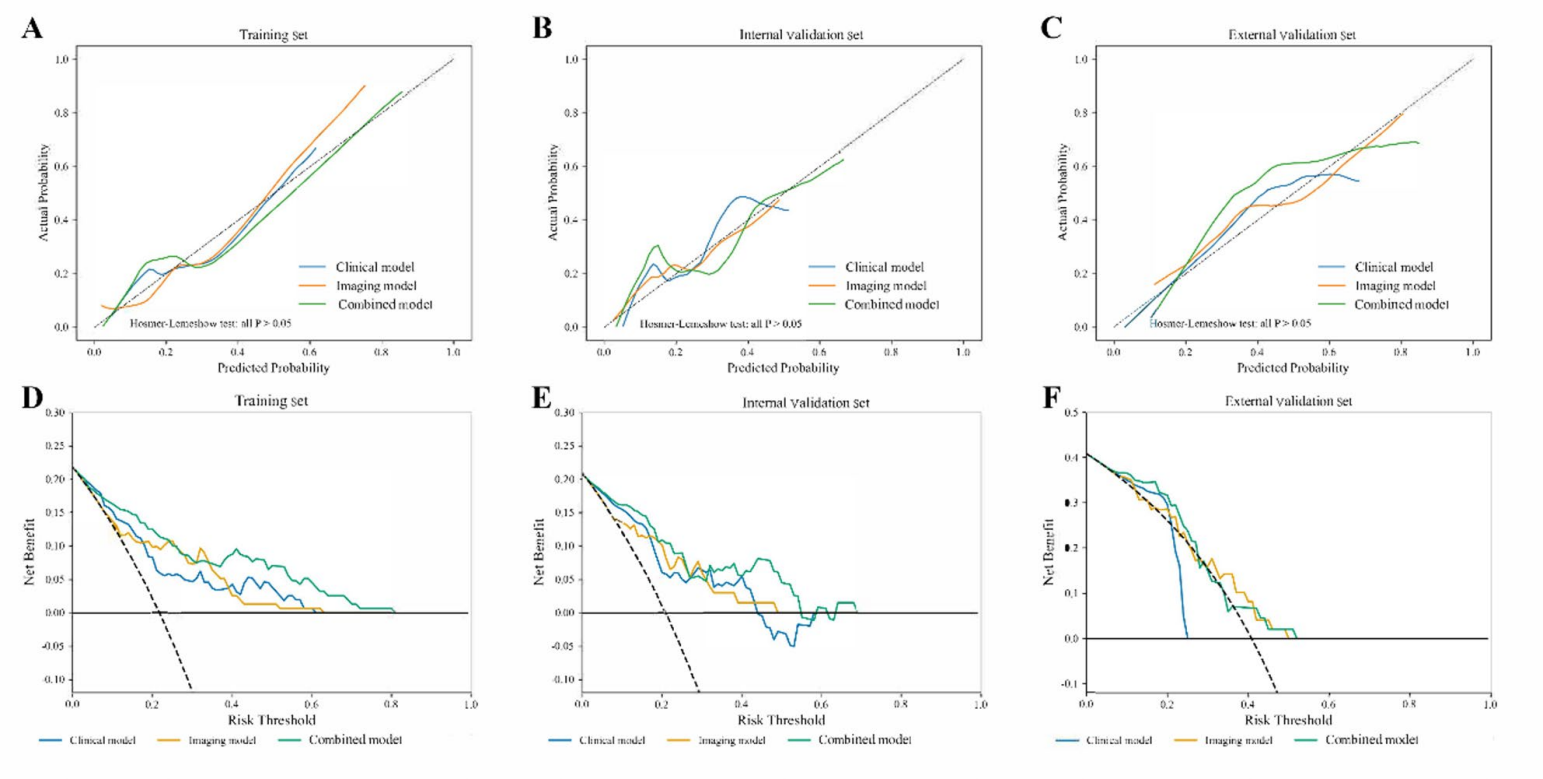
Calibration and Decision Curve Analyses of the Three Models Across All Sets. (**A**–**C**) Calibration curves for the clinical, imaging, and combined models in the training, internal validation, and external validation sets, respectively, showing that the combined model yielded the best overall calibration. (**D**–**F**) Decision curve analyses in the corresponding datasets demonstrated that the combined model consistently provided the greatest net benefit across a wide range of threshold probabilities

Calibration curve analyses indicated that the combined model exhibited good agreement between predicted and observed outcomes (Fig. 4A and C). Decision curve analysis further demonstrated that the combined model provided the highest net clinical benefit across most threshold probabilities (Fig. 4D and F).

### SHAP interpretability analysis

**Fig. 5:**
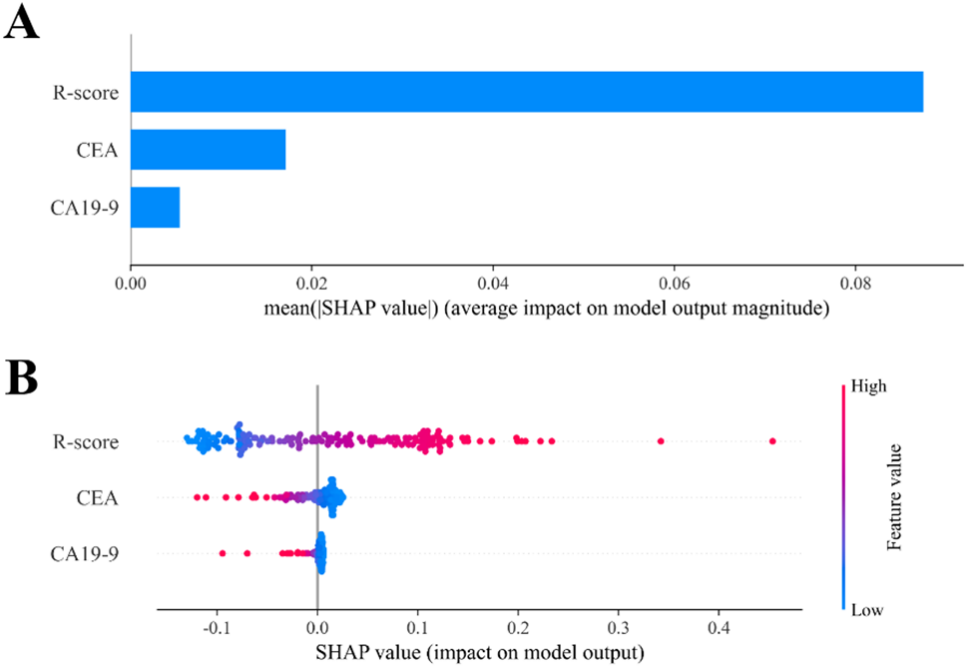
SHAP analysis of the combined model. (**A**) Mean SHAP values of each feature show that R-score contributed most to model predictions. (**B**) SHAP scatter plot demonstrates the distribution of feature impacts, with higher R-score values linked to higher predicted probabilities of treatment response

SHAP interpretability analysis revealed that the R-score was the most influential feature in the combined model, contributing substantially more to the model output than CEA and CA19-9 (Fig. 5A).

In the SHAP scatter plot, higher values of R-score (in red) were positively associated with a greater predicted probability of response to nCRT, further confirming its dominant role in model decision-making (Fig. 5B).

## Discussion

Accurately predicting the efficacy of nCRT in patients with LARC is essential for implementing individualized treatment strategies. In this study, we developed an interpretable XGBoost-based machine learning model (combined model) that integrates contrast-enhanced CT radiomics features with clinical biomarkers such as CEA and CA19-9. The combined model achieved AUCs of 0.844 and 0.800 in the internal and external validation sets, respectively, outperforming models based solely on imaging or clinical features, and demonstrating promising generalizability and clinical applicability.

While conventional imaging modalities such as MRI and CT can provide information on tumor staging, volumetric changes, and lymph node status [14, 15], their predictive accuracy remains limited due to subjectivity, interobserver variability, and delayed assessment. In contrast, our study utilized a machine learning framework to synergistically combine quantitative CT-based radiomic features with clinical biomarkers (CEA and CA19-9), thereby constructing a multidimensional and interpretable predictive model. Radiomics enables high-throughput extraction of subvisual tumor characteristics, quantifying tumor morphology and texture heterogeneity beyond what is appreciable by human observers [13, 16]. Previous studies have similarly explored advanced quantitative metrics to characterize this complexity. For instance, fractal dimension analysis on pretreatment CT has been identified as a promising biomarker for predicting pathological complete response [17], while MRI-based habitat analysis has demonstrated the value of partitioning tumors into distinct subregions to quantify spatial intratumoral heterogeneity [18]. Inspired by these recent advances, based on LASSO regression, we selected 10 features to construct the R-score, which, when combined with clinical variables, formed a comprehensive feature space representing both tumor-intrinsic heterogeneity and host-related systemic biology [19–23]. This integrative approach likely contributed to the model’s robust predictive performance across all datasets (training set AUC = 0.858; internal validation set AUC = 0.844; external validation set AUC = 0.800).

To enhance model transparency and clinical acceptance, we further incorporated SHAP to interpret the model’s predictions [24, 25]. SHAP analysis revealed that the R-score was the most important predictor in the combined model, followed by CEA and CA19-9. The SHAP scatter plot showed that higher R-score values (in red) were positively associated with higher predicted probabilities of nCRT response, underscoring the dominant role of radiomics in decision-making. The identified predictors are also biologically plausible. For instance, the predictive value of baseline CEA level aligns with previous evidence linking elevated CEA to tumor aggressiveness and metastatic potential [26], further reinforcing the validity of our model.

Our interpretable model offers several practical advantages for clinical translation. First, the nomogram and machine learning interface support intuitive, real-time decision-making without requiring specialized computational expertise. Second, its consistent performance in the external validation cohort (AUC = 0.800) indicates potential for broader institutional generalization. Most importantly, decision curve analysis confirmed that the combined model yielded the highest net clinical benefit across a wide range of clinically relevant thresholds (0.05–0.55), facilitating rational stratification of patients into those suitable for organ-preservation strategies (predicted responders) versus those requiring intensified treatment (predicted non-responders). This addresses a critical gap in current LARC management, where overtreatment of responders and undertreatment of resistant disease remain major concerns.

### Limitations

Several limitations of this study should be noted. First, this was a retrospective study, and selection bias may exist due to institutional differences in the external validation cohort. Second, tumor ROI delineation was manually performed, introducing potential observer variability; future work should explore semi- or fully automated segmentation methods to improve reproducibility. Third, this study was limited by the exclusive use of CT data, as MRI data were not available for all patients. Future studies incorporating multimodal MRI features are warranted to enable direct modality comparison and to assess potential performance gains from multimodal integration. Fourth, the external validation set included fewer than 50 patients, which may limit the statistical power and generalizability of the findings. Further validation in larger multicenter cohorts is needed. Fifth, although our model demonstrated good performance in predicting short-term treatment response, its association with long-term outcomes such as disease-free survival and overall survival remains unclear. Prospective studies with follow-up data are necessary to assess its prognostic value. Finally, while our model combined radiomics and conventional clinical features, it did not incorporate emerging biomarkers such as circulating tumor DNA or immune profiling [27], which may further enhance predictive performance. Fourth, the current model has not yet been implemented as an interactive clinical tool; development of a web-based or PACS-integrated decision support system would facilitate clinical adoption and multicenter deployment.

## Conclusion

In conclusion, this study developed and validated an interpretable machine learning model integrating enhanced CT-based radiomics and clinical biomarkers for predicting treatment response to nCRT in patients with LARC. The model demonstrated robust and consistent performance across multiple datasets, and SHAP-based interpretability enhanced its clinical acceptability. Further validation in large-scale prospective multicenter cohorts is warranted to confirm its clinical utility and promote real-world application.

## Supplementary Information

Below is the link to the electronic supplementary material.

Supplementary Material 1

## Data Availability

The dataset analyzed during the current study is available from the corresponding author on reasonable request.

## Abbreviations

AUC: Area Under the Receiver Operating Characteristic Curve
LARC: locally advanced rectal cancer
nCRT: neoadjuvant chemoradiotherapy
OR: odds ratio
CI: confidence interval

## References

[1] Liu S, Jiang T, Xiao L, Yang S, Liu Q, Gao Y, Chen G, Xiao W. Total Neoadjuvant Therapy (TNT) versus Standard Neoadjuvant Chemoradiotherapy for Locally Advanced Rectal Cancer: A Systematic Review and Meta-Analysis. Oncologist. 2021;26(9):e1555–66. 33987952 10.1002/onco.13824 PMC8417863

[2] Abedizadeh R, Majidi F, Khorasani HR, Abedi H, Sabour D. Colorectal cancer: a comprehensive review of carcinogenesis, diagnosis, and novel strategies for classified treatments. Cancer Metastasis Rev. 2024;43(2):729–53. 38112903 10.1007/s10555-023-10158-3

[3] Nelson VM, Benson AB. 3rd: Pathological complete response after neoadjuvant therapy for rectal cancer and the role of adjuvant therapy. Curr Oncol Rep. 2013;15(2):152–61. 23381584 10.1007/s11912-013-0297-5

[4] Koukourakis IM, Kouloulias V, Tiniakos D, Georgakopoulos I, Zygogianni A. Current status of locally advanced rectal cancer therapy and future prospects. Crit Rev Oncol Hematol. 2023;186:103992. 37059276 10.1016/j.critrevonc.2023.103992

[5] Teng H, Wang Y, Sui X, Fan J, Li S, Lei X, Shi C, Sun W, Song M, Wang H, et al. Gut microbiota-mediated nucleotide synthesis attenuates the response to neoadjuvant chemoradiotherapy in rectal cancer. Cancer Cell. 2023;41(1):124–e138126. 36563680 10.1016/j.ccell.2022.11.013

[6] Saraf A, Roberts HJ, Wo JY, Parikh AR. Optimal Neoadjuvant Strategies for Locally Advanced Rectal Cancer by Risk Assessment and Tumor Location. J Natl Compr Canc Netw. 2022;20(10):1177–84. 36240854 10.6004/jnccn.2022.7061

[7] Bedin C, Crotti S, D’Angelo E, D’Aronco S, Pucciarelli S, Agostini M. Circulating Biomarkers for Response Prediction of Rectal Cancer to Neoadjuvant Chemoradiotherapy. Curr Med Chem. 2020;27(25):4274–94. 31060482 10.2174/0929867326666190507084839

[8] Zhang YX, Liu HY, Jiang B, Wang WY, Wang HB, Lu YJ. Stepwise neoadjuvant chemoradiotherapy in the management of mid-low locally advanced rectal cancer. Eur J Surg Oncol. 2020;46(3):410–4. 31627933 10.1016/j.ejso.2019.10.012

[9] Jayaprakasam VS, Alvarez J, Omer DM, Gollub MJ, Smith JJ, Petkovska I. Watch-and-Wait Approach to Rectal Cancer: The Role of Imaging. Radiology. 2023;307(1):e221529. 36880951 10.1148/radiol.221529 PMC10068893

[10] Jayaprakasam VS, Ince S, Suman G, Nepal P, Hope TA, Paspulati RM, Fraum TJ. PET/MRI in colorectal and anal cancers: an update. Abdom Radiol (NY). 2023;48(12):3558–83. 37062021 10.1007/s00261-023-03897-y

[11] De Felice F, Crocetti D, Petrucciani N, Belgioia L, Sapienza P, Bulzonetti N, Marampon F, Musio D, Tombolini V. Treatment in locally advanced rectal cancer: a machine learning bibliometric analysis. Th Adv Gastroenterol. 2021;14:17562848211042170. 10.1177/17562848211042170 PMC8521411 34671421

[12] Stanzione A, Verde F, Romeo V, Boccadifuoco F, Mainenti PP, Maurea S. Radiomics and machine learning applications in rectal cancer: Current update and future perspectives. World J Gastroenterol. 2021;27(32):5306–21. 34539134 10.3748/wjg.v27.i32.5306 PMC8409167

[13] Liu X, Zhang D, Liu Z, Li Z, Xie P, Sun K, Wei W, Dai W, Tang Z, Ding Y, et al. Deep learning radiomics-based prediction of distant metastasis in patients with locally advanced rectal cancer after neoadjuvant chemoradiotherapy: A multicentre study. EBioMedicine. 2021;69:103442. 34157487 10.1016/j.ebiom.2021.103442 PMC8237293

[14] Feng L, Liu Z, Li C, Li Z, Lou X, Shao L, Wang Y, Huang Y, Chen H, Pang X, et al. Development and validation of a radiopathomics model to predict pathological complete response to neoadjuvant chemoradiotherapy in locally advanced rectal cancer: a multicentre observational study. Lancet Digit Health. 2022;4(1):e8–17. 34952679 10.1016/S2589-7500(21)00215-6

[15] Wu X, Wang J, Chen C, Cai W, Guo Y, Guo K, Chen Y, Shi Y, Chen J, Lin X, et al. Integration of Deep Learning and Sub-regional Radiomics Improves the Prediction of Pathological Complete Response to Neoadjuvant Chemoradiotherapy in Locally Advanced Rectal Cancer Patients. Acad Radiol. 2025;32(6):3384–96. 39809603 10.1016/j.acra.2024.12.049

[16] Horvat N, Papanikolaou N, Koh DM. Radiomics Beyond the Hype: A Critical Evaluation Toward Oncologic Clinical Use. Radiol Artif Intell. 2024;6(4):e230437. 38717290 10.1148/ryai.230437 PMC11294952

[17] Tochigi T, Kamran SC, Parakh A, Noda Y, Ganeshan B, Blaszkowsky LS, Ryan DP, Allen JN, Berger DL, Wo JY, et al. Response prediction of neoadjuvant chemoradiation therapy in locally advanced rectal cancer using CT-based fractal dimension analysis. Eur Radiol. 2022;32(4):2426–36. 34643781 10.1007/s00330-021-08303-z

[18] Chen Q, Zhang Q, Li Z, Zhang S, Xia Y, Wang H, Lu Y, Zheng A, Shao C, Shen F. MRI-based habitat analysis for pathologic response prediction after neoadjuvant chemoradiotherapy in rectal cancer: a multicenter study. Eur Radiol 2025. 10.1007/s00330-025-11997-0 40981989

[19] Chang KJ, Kim DH, Lalani TK, Paroder V, Pickhardt PJ, Shaish H, Bates DDB. Radiologic T staging of colon cancer: renewed interest for clinical practice. Abdom Radiol (NY). 2023;48(9):2874–87. 37277570 10.1007/s00261-023-03904-2

[20] Huang D, Lin Q, Song J, Xu B. Prognostic Value of Pretreatment Serum CA199 in Patients with Locally Advanced Rectal Cancer Treated with CRT Followed by TME with Normal Pretreatment Carcinoembryonic Antigen Levels. Dig Surg. 2021;38(1):24–9. 33171467 10.1159/000508442

[21] Huang Q, Qin H, Xiao J, He X, Xie M, He X, Yao Q, Lan P, Lian L. Association of tumor differentiation and prognosis in patients with rectal cancer undergoing neoadjuvant chemoradiation therapy. Gastroenterol Rep (Oxf). 2019;7(4):283–90. 31413836 10.1093/gastro/goy045 PMC6688738

[22] Karahacioglu D, Taskin OC, Esmer R, Armutlu A, Saka B, Ozata IH, Rencuzogullari A, Bugra D, Balik E, Adsay V, et al. Performance of CT in the locoregional staging of colon cancer: detailed radiology-pathology correlation with special emphasis on tumor deposits, extramural venous invasion and T staging. Abdom Radiol (NY). 2024;49(6):1792–804. 38446179 10.1007/s00261-024-04203-0

[23] Lin Q, Wu HJ, Song QS, Tang YK. CT-based radiomics in predicting pathological response in non-small cell lung cancer patients receiving neoadjuvant immunotherapy. Front Oncol. 2022;12:937277. 36267975 10.3389/fonc.2022.937277 PMC9577189

[24] Li J, Liu S, Hu Y, Zhu L, Mao Y, Liu J. Predicting Mortality in Intensive Care Unit Patients With Heart Failure Using an Interpretable Machine Learning Model: Retrospective Cohort Study. J Med Internet Res. 2022;24(8):e38082. 35943767 10.2196/38082 PMC9399880

[25] Musolf AM, Holzinger ER, Malley JD, Bailey-Wilson JE. What makes a good prediction? Feature importance and beginning to open the black box of machine learning in genetics. Hum Genet. 2022;141(9):1515–28. 34862561 10.1007/s00439-021-02402-z PMC9360120

[26] Campos-da-Paz M, Dórea JG, Galdino AS, Lacava ZGM, de Fatima Menezes Almeida Santos M. Carcinoembryonic Antigen (CEA) and Hepatic Metastasis in Colorectal Cancer: Update on Biomarker for Clinical and Biotechnological Approaches. Recent Pat Biotechnol. 2018;12(4):269–79. 30062978 10.2174/1872208312666180731104244

[27] Wei Q, Chen Z, Tang Y, Chen W, Zhong L, Mao L, Hu S, Wu Y, Deng K, Yang W, et al. External validation and comparison of MR-based radiomics models for predicting pathological complete response in locally advanced rectal cancer: a two-centre, multi-vendor study. Eur Radiol. 2023;33(3):1906–17. 36355199 10.1007/s00330-022-09204-5

