# Supplementary material for: Interpretable machine learning models based on CT radiomics for predicting chemoradiotherapy response in rectal cancer
Source: BMC Med Imaging. 2026 Mar 20;26:218. doi: 10.1186/s12880-026-02269-4 (PMC13127060; doi:10.1186/s12880-026-02269-4)
Supplement: Supplementary file 1 — Supplementary Material 1 [file 12880_2026_2269_MOESM1_ESM.docx]

**Supplementary Material**

**Table S1. Comparison of Clinical Characteristics Between Responders and Non-Responders in the Training Set**

| **Characteristics** | **Training Set (*n* = 156)** | | ***P* Value** |
| --- | --- | --- | --- |
|  | **Responders**  **(*n* = 34)** | **Non-Responders**  **(*n* = 122)** |  |
| **Age (years)** | 56.68 ± 9.71 | 57.35 ± 10.45 | 0.735 |
| **BMI** | 23.53 ± 2.66 | 22.92 ± 3.11 | 0.173 |
| **Sex** |  |  | 0.324 |
| Male | 30 (88.2) | 96 (78.7) |  |
| Female | 4 (11.8) | 26 (21.3) |  |
| **CA19-9 (kU/L)** | 9.68 ± 6.95 | 30.07 ± 69.92 | < 0.001 |
| **CEA (ng/mL)** | 3.39 ± 2.59 | 7.43 ± 9.18 | 0.019 |
| **T stage** |  |  | < 0.001 |
| T2 | 4 (11.8) | 0 (0.0) |  |
| T3 | 23 (67.6) | 73 (59.8) |  |
| T4 | 7 (20.6) | 49 (40.2) |  |
| **N stage** |  |  | 0.357 |
| N0 | 11 (32.4) | 26 (21.3) |  |
| N1 | 14 (41.2) | 46 (37.7) |  |
| N2 | 8 (23.5) | 40 (32.8) |  |
| N3 | 1 (2.9) | 10 (8.2) |  |
| **Tumor location** |  |  | 0.479 |
| Low | 24 (70.6) | 73 (59.8) |  |
| Mid | 9 (26.5) | 46 (37.7) |  |
| High | 1 (2.9) | 3 (2.5) |  |
| **Tumor-differentiation grade** |  |  | < 0.001 |
| Poorly | 25 (73.5) | 14 (11.5) |  |
| Moderately | 9 (26.5) | 92 (75.4) |  |
| Well | 0 (0.0) | 16 (13.1) |  |
| Note.—Except where indicated, data are numbers of patients, with percentages in parentheses. | | | |

**Table S2. Comparison of Clinical Characteristics Between** **Responders and Non-Responders in the Internal Validation Set**

| **Characteristics** | **Internal Validation Set (*n* = 67)** | | ***P* Value** |
| --- | --- | --- | --- |
|  | **Responders**  **(*n* = 14)** | **Non-Responders**  **(*n* = 53)** |  |
| **Age (years)** | 55.00 ± 12.28 | 57.11 ± 11.17 | 0.517 |
| **BMI** | 25.31 ± 2.78 | 24.12 ± 2.75 | 0.103 |
| **Sex** |  |  | 0.727 |
| Male | 10 (71.4) | 41 (77.4) |  |
| Female | 4 (28.6) | 12 (22.6) |  |
| **CA19-9 (kU/L)** | 10.61 ± 5.80 | 19.60 ± 17.11 | 0.094 |
| **CEA (ng/mL)** | 2.40 ± 1.27 | 7.36 ± 6.92 | 0.005 |
| **T stage** |  |  | 0.081 |
| T2 | 1 (7.1) | 0 (0.0) |  |
| T3 | 10 (71.4) | 32 (60.4) |  |
| T4 | 3 (21.4) | 21 (39.6) |  |
| **N stage** |  |  | 0.073 |
| N0 | 2 (14.3) | 8 (15.1) |  |
| N1 | 7 (50.0) | 17 (32.1) |  |
| N2 | 3 (21.4) | 27 (50.9) |  |
| N3 | 2 (14.3) | 1 (1.9) |  |
| **Tumor location** |  |  | 0.120 |
| Low | 5 (35.7) | 35 (66.0) |  |
| Mid | 7 (50.0) | 14 (26.4) |  |
| High | 2 (14.3) | 4 (7.5) |  |
| **Tumor-differentiation grade** |  |  | < 0.001 |
| Poorly | 11 (78.6) | 8 (15.1) |  |
| Moderately | 1 (7.1) | 41 (77.4) |  |
| Well | 2 (14.3) | 4 (7.5) |  |
| Note.—Except where indicated, data are numbers of patients, with percentages in parentheses. | | | |

**Table S3. Comparison of Clinical Characteristics Between Responders and Non-Responders in the External Validation Set**

| **Characteristics** | **External Validation Set (*n* = 49)** | | ***P* Value** |
| --- | --- | --- | --- |
|  | **Responders**  **(*n* = 20)** | **Non-Responders**  **(*n* = 29)** |  |
| **Age (years)** | 55.45 ± 11.17 | 55.07 ± 7.91 | 0.889 |
| **BMI** | 24.09 ± 2.26 | 23.40 ± 2.91 | 0.375 |
| **Sex** |  |  | 0.923 |
| Male | 14 (70.0) | 21 (72.4) |  |
| Female | 6 (30.0) | 8 (27.6) |  |
| **CA19-9 (kU/L)** | 10.03 ± 6.61 | 24.47 ± 17.78 | 0.021 |
| **CEA (ng/mL)** | 3.16 ± 2.31 | 7.83 ± 6.18 | 0.013 |
| **T stage** |  |  | 0.220 |
| T2 | 0 (0.0) | 2 (6.9) |  |
| T3 | 14 (70.0) | 14 (48.3) |  |
| T4 | 6 (30.0) | 13 (44.8) |  |
| **N stage** |  |  | 0.789 |
| N0 | 2 (10.0) | 4 (13.8) |  |
| N1 | 8 (40.0) | 9 (31.0) |  |
| N2 | 10 (50.0) | 16 (55.2) |  |
| N3 | 0 | 0 |  |
| **Tumor location** |  |  | 0.711 |
| Low | 10 (50.0) | 12 (41.4) |  |
| Mid | 8 (40.0) | 15 (51.7) |  |
| High | 2 (10.0) | 2 (6.9) |  |
| **Tumor-differentiation grade** |  |  | 0.215 |
| Poorly | 2 (10.0) | 5 (17.2) |  |
| Moderately | 13 (65.0) | 20 (69.0) |  |
| Well | 5 (25.0) | 4 (13.8) |  |
| Note.—Except where indicated, data are numbers of patients, with percentages in parentheses. | | | |


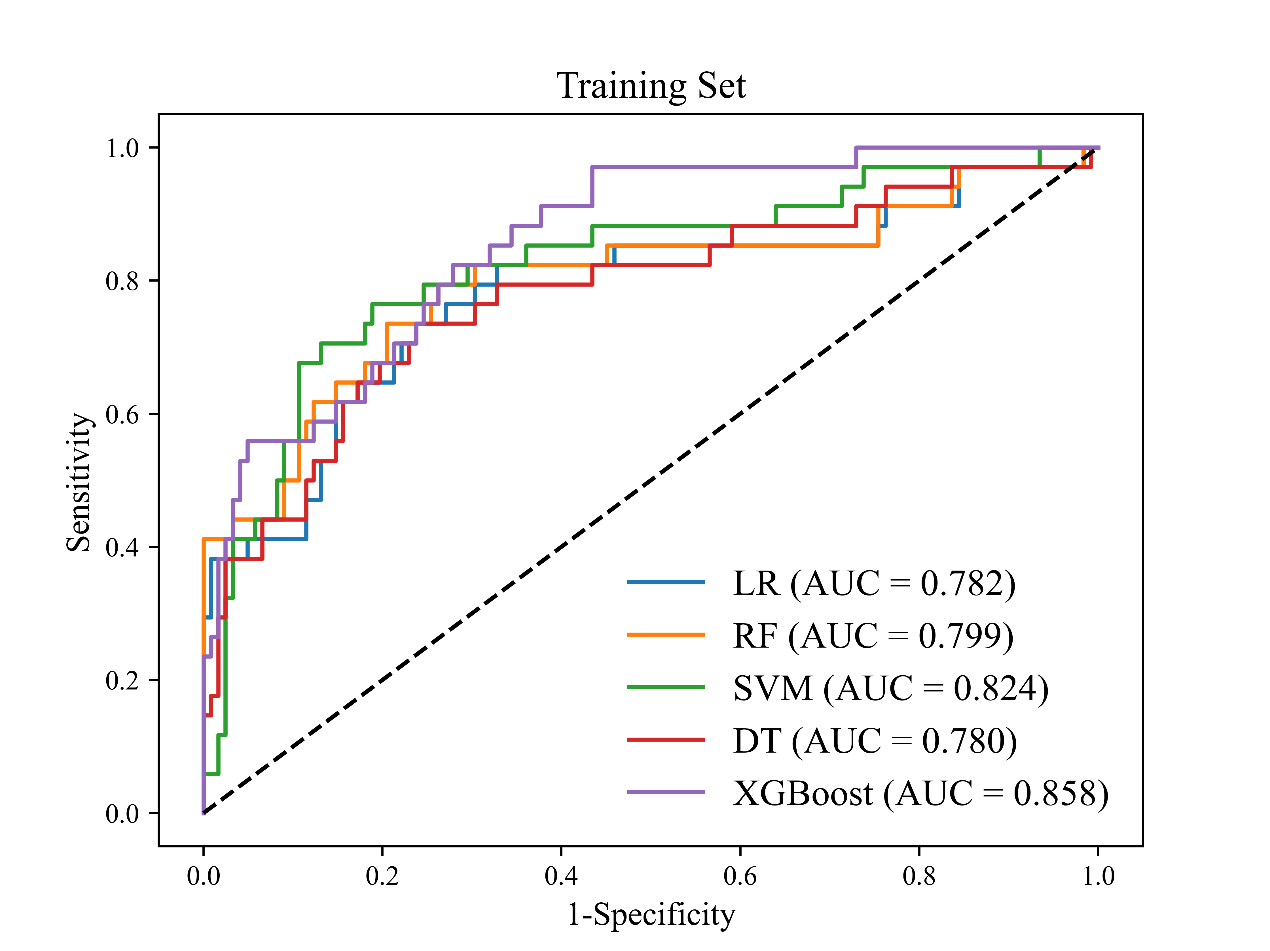


**Figure S1.** ROC Curves of Five Machine Learning Models in the Training Set. LR = logistic regression, RF = random forest, SVM = support vector machine, DT = decision tree.
